# Glucocorticoid receptor in astrocytes regulates midbrain dopamine neurodegeneration through connexin hemichannel activity

**DOI:** 10.1038/s41418-018-0150-3

**Published:** 2018-07-13

**Authors:** Layal Maatouk, Chenju Yi, Maria-Angeles Carrillo-de Sauvage, Anne-Claire Compagnion, Stéphane Hunot, Pascal Ezan, Etienne C. Hirsch, Annette Koulakoff, Frank W Pfrieger, François Tronche, Luc Leybaert, Christian Giaume, Sheela Vyas

**Affiliations:** 1 Institute of Biology Paris Seine, Team Gene Regulation and Adaptive Behaviors, Department of Neurosciences Paris Seine, Sorbonne Université, CNRS UMR 8246, INSERM U1130, 9 Quai Saint Bernard, F-75005 Paris, France; 2 CIRB, UMR CNRS 7241/ INSERM 1050, Collège de France, Paris, France; 3 CEA, DRF, MIRCen, Université Paris-Sud, CNRS UMR 9199, Neurodegenerative Diseases Laboratory, F-92260 Fontenay-aux-Roses, France; 4 Inserm UMRS 1127, CNRS UMR 7225, Sorbonne Université, Institut du Cerveau et de la Moelle Epinière (ICM), F-75013 Paris, France; 5 Institute of Cellular and Integrative Neurosciences, CNRS UPR 3212, University of Strasbourg, Strasbourg, France; 6 Physiology group, Department of Basic Medical Sciences, Faculty of Medicine and Health Sciences, Ghent University, Ghent, Belgium

**Keywords:** Outcomes research, Glial biology

## Abstract

The precise contribution of astrocytes in neuroinflammatory process occurring in Parkinson’s disease (PD) is not well characterized. In this study, using GR^Cx30CreERT2^ mice that are conditionally inactivated for glucocorticoid receptor (GR) in astrocytes, we have examined the actions of astrocytic GR during dopamine neuron (DN) degeneration triggered by the neurotoxin 1-methyl-4-phenyl-1,2,3,6-tetrahydropyridine (MPTP). The results show significantly augmented DN loss in GR^Cx30CreERT2^ mutant mice in substantia nigra (SN) compared to controls. Hypertrophy of microglia but not of astrocytes was greatly enhanced in SN of these astrocytic GR mutants intoxicated with MPTP, indicating heightened microglial reactivity compared to similarly-treated control mice. In the SN of GR astrocyte mutants, specific inflammation-associated transcripts *ICAM-1*, *TNF*-α and *Il-1β* as well as TNF-α protein levels were significantly elevated after MPTP neurotoxicity compared to controls. Interestingly, this paralleled increased connexin hemichannel activity and elevated intracellular calcium levels in astrocytes examined in acute midbrain slices from control and mutant mice treated with MPP+ . The increased connexin-43 hemichannel activity was found in vivo in MPTP-intoxicated mice. Importantly, treatment of MPTP-injected GR^Cx30CreERT2^ mutant mice with TAT-Gap19 peptide, a specific connexin-43 hemichannel blocker, reverted both DN loss and microglial activation; in wild-type mice there was partial but significant survival effect. In the SN of post-mortem PD patients, a significant decrease in the number of astrocytes expressing nuclear GR was observed, suggesting the participation of astrocytic GR deregulation of inflammatory process in PD. Overall, these data provide mechanistic insights into GR-modulated processes in vivo, specifically in astrocytes, that contribute to a pro-inflammatory state and dopamine neurodegeneration in PD pathology.

## Introduction

Reactive glial cells are key elements in the physiopathology of Parkinson’s disease (PD) that is characterized by progressive degeneration of dopaminergic neurons (DNs) in the substantia nigra (SN). This loss of DNs, along with a significant depletion of dopamine in nigrostriatal dopaminergic terminals, is at the origin of cardinal motor symptoms of PD including akinesia, tremor at rest and rigidity [1]. Although both microglia and astrocytes play a role in neurodegeneration through secretion of potent inflammatory mediators such as TNF-α or IL-1β, they most likely differ in their contribution to PD pathology. Microglial activation in PD patients is well documented by PET imaging and analysis of post-mortem tissues [2, 3]. However, given the lack of PET imaging markers specific for reactive astrocytes, post-mortem studies of PD relying on immunohistochemical analyses of astrocytes have yielded conflicting results. Thus, GFAP labeling of astrocytes revealed either no change [4, 5] or increased numbers GFAP-positive astrocytes [6] or astrocyte hypertrophy [7]. Nevertheless, post-mortem studies indicate the involvement of astrocytes in PD pathology. They revealed i) accumulation of α-synuclein in astrocytes [8, 9] and its correlation with the extent of severity of nigral DN loss [10]; ii) fewer glutathione peroxidase (enzyme involved in hydrogen peroxide scavenging) positive astrocytes in SN of PD patients [6]; iii) strong expression of I-CAM and myeloperoxidase in astrocytes of SN [11] and finally iv) increased S100β expression in astrocytes [12], which may augment the immune responses through activation of RAGE and TNF-α receptors.

In pre-clinical models of PD, there is evidence of reactive astrocytes participating in the pro-inflammatory responses that lead to degeneration of DNs in SN [11, 13, 14]. The astrocyte-specific mechanisms, which control these inflammatory processes, are not well understood. Nuclear hormone receptors are known to regulate inflammation through gene transcription and represent key candidates [15]. There is evidence for a role of glucocorticoids acting through glucocorticoid receptors (GRs), archetypal nuclear receptors, in the pathophysiology of PD. For instance, basal plasma cortisol levels are significantly elevated in PD patients, suggesting a deregulated hypothalamo-pituitary-adrenal axis, which is known to affect GR activity [16]. Therefore immune responses under GR control may also be deregulated in PD. We previously showed that total GR levels are reduced in SN of post-mortem PD, as well microglial/macrophagic GR plays a crucial role in the survival of DNs following treatment with Parkinsonian neurotoxin MPTP (which induces selective degeneration of DNs by targeting DNs leading to gliosis) [17].

To understand the role of GR in astrocytes during dopamine neurodegeneration, we inactivated GR gene in protoplasmic astrocytes by crossing BAC-Tg (Cx30-CreERT2) mice [18] with GR loxP/loxP mice [19]. We show that loss of astrocyte GR signaling increases the expression of specific inflammatory genes in SN, induces microglial activation and enhances degeneration of DNs following MPTP treatment. Importantly, connexin-mediated hemichannel activity plays a crucial role in these actions of astrocytic GR. Finally, analysis of astrocytes in SN of PD postmortem brain samples revealed a significant decrease in number of astrocytes expressing GR suggesting, together with animal data, that compromised GR signaling most likely contributes to neurodegeneration in PD.

## Results

### Evaluation of Cre expression and recombination efficiency for GR specifically in astrocytes of GR^Cx30CreERT2^ mice

**Fig. 1:**
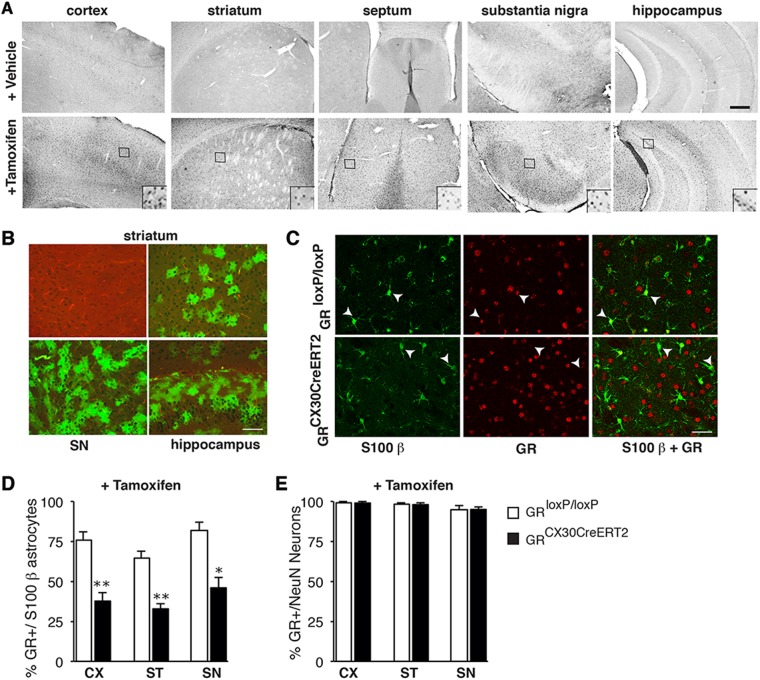
Tamoxifen-dependent Cre expression, recombination and determination of GR deletion in GR^Cx30CreERT2^ mice. **a** Representative panels showing Cre immunohistochemistry in GR^Cx30CreERT2^ mice injected with either vehicle or tamoxifen, in cortex (cx), striatum, septum, substantia nigra (SN) and hippocampus. The small squares in tamoxifen-positive images are magnified in upper panels to show Cre recombinase. Scale bar, 200 μm. **b** Representative images showing mTomato (red) staining after vehicle injection and appearance of mGFP expression in astrocytes of striatum, substantia nigra (SN) and hippocampus after tamoxifen injections in Cx30CreERT2:mT/mG mice. Scale bar, 100 μm. **c** Representative images showing double labeling of GR (red) and S100β (green) in cortex of GR^loxp/loxP^ controls and GR^Cx30CreERT2^ mice injected with tamoxifen. Scale bar, 5 μm. **d**, **e** Quantification of the percentage of S100β + astrocytes co-labeled with GR and (**d**) and NeuN + neurons expressing GR (**e**) in cortex (Cx), striatum (ST) and substantia nigra (SN) of controls and GR^Cx30CreERT2^ mutant mice injected with tamoxifen. *p < 0.05; **p < 0.01, mutant vs control. Error bars represent SEM. *n* = 4–5/group

Widespread Cre expression evident in the cortex, septum, SN, striatum and hippocampus was observed only in GR^Cx30CreERT2^ mutant mice injected with tamoxifen but not with vehicle (Fig. 1a). The tamoxifen-induced Cre recombination was verified in mT/mG^Cx30CreERT2^ reporter mice, where Cre-mediated excision induces expression of GFP. GFP + cells were found in all brain regions examined (Fig. 1b). The efficacy of GR inactivation by Cre recombination in the astrocytes of GR^Cx30CreERT2^ mutants was determined by co-labeling of GR and S100β with quantification of GR + astrocytes in control and mutant mice. In controls, on average 75% of S100β-positive astrocytes expressed GR compared to only 39% in the mutants. The Cre recombination efficiency varied between 51% in the cortex, 49% in the striatum and 44% in the SN (Fig. 1c, d). These values are similar to those reported in Cx30 mice using a different reporter [18] and in GFAP-CreERT2 conditional astrocyte mice [20]. The Cre recombination in GR^Cx30CreERT2^ mutant mice was specific to astrocytes as GR expression in NeuN + neurons of cortex, striatum and SN was unaffected (Fig. 1e).

### Loss of astrocytic GR increases susceptibility of substantia nigra DNs to MPTP toxicity

**Fig. 2:**
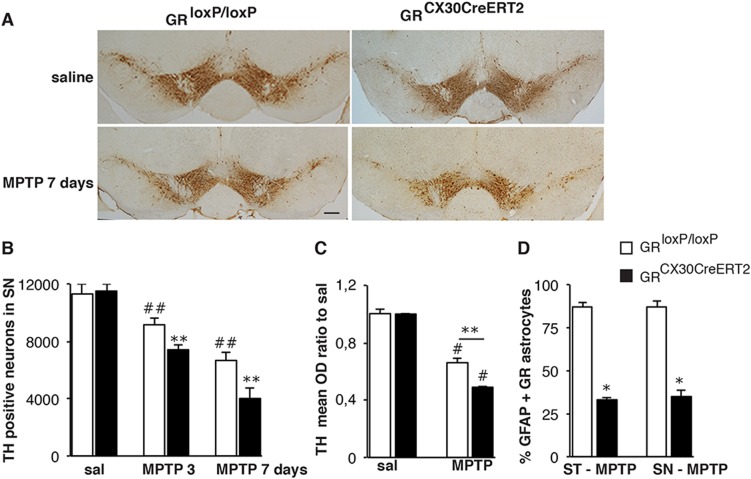
Loss of astrocytic GR exacerbates MPTP-induced degeneration of DNs. **a** TH immunohistochemistry in SN of control and GR^Cx30CreERT2^ mice 7 days after acute MPTP intoxication. Scale bar, 100 μm. **b** Quantification of TH + neurons in SN 3 and 7 days after MPTP treatment, showing a significant reduction in GR^Cx30CreERT2^ mutants compared with controls. #*p* < 0.05; ##*p* < 0.01 saline vs MPTP; *p *<* 0.05; **p < 0.01 controls vs mutants, error bars represent SEM, post-hoc Bonferroni/Dunn test, *n* = 5 mice/group. **c** Optical density of TH immunoreactivity in striatum normalized to saline-treated mice 3 days after MPTP intoxication. MPTP decreases TH density more strongly in mutants compared to control animals indicative of further loss of DN nerve terminals. #*p* < 0.05 saline vs MPTP. **p < 0.01 controls vs mutants, error bars represent SEM. *n* = 5 mice/group. **d** Percentage of GFAP + astrocytes expressing GR in striatum (ST) and SN in control and mutant GR^Cx30CreERT2^ mice 3 days following MPTP intoxication. *p < 0.05 controls vs mutants, *n* = 5 mice/group

To study the contribution of astrocytic GR to neurodegeneration in PD, tamoxifen pre-treated control and GR^Cx30CreERT2^ mutant mice were injected with either saline or MPTP. DNs in SN were quantified following immunohistochemistry (IHC) for tyrosine hydroxylase (TH). MPTP triggered DN loss of around 20% in controls at 3 days and 41% at 7 days whereas in the mutants the DN loss was significantly more robust averaging 30 and 62% at 3 and 7 days respectively (Fig. 2a, b). This finding indicated that astrocytic GR protects DNs against MPTP-mediated nigrostriatal injury. This was further supported by striatal TH IHC of dopaminergic nerve terminals at 3 days. Optical density analysis showed larger decrease in mutants (51%) compared to controls (34%) after MPTP treatment (Fig. 2c). Previously, we reported increased density of GR-positive microglial nuclei upon a surge in GC levels during MPTP intoxication [17]. We quantified GR + GFAP + astrocytes in the striatum and SN 3 days following MPTP treatment. The results showed 87% of GFAP + astrocytes of SN and striatum expressed GR in controls whereas in mutants only 33% of GFAP + astrocytes showed GR labeling, indicating a Cre recombination of 62% (Fig. 2d).

### Microglial but not astrocyte reactivity is augmented in GR^Cx30CreERT2^ mutant mice following MPTP intoxication

**Fig. 3:**
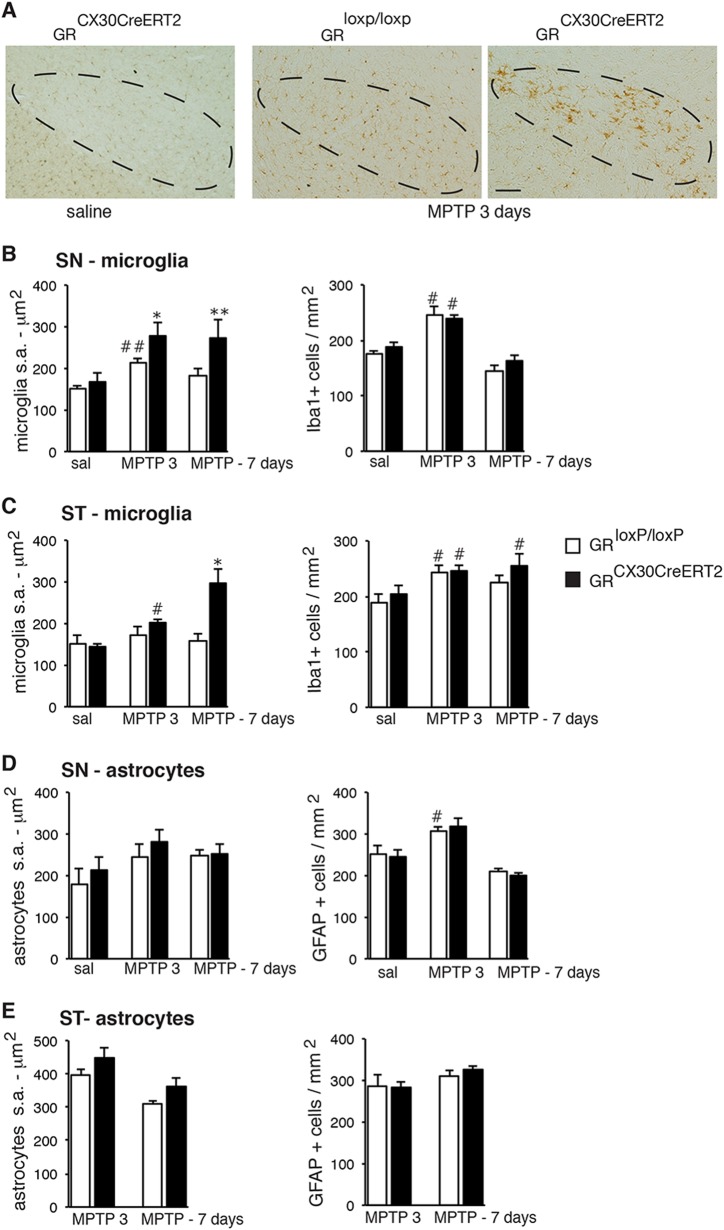
Analyses of glial reactivity resulting from MPTP intoxication in control and astrocytic GR mutant mice. **a** Immunohistochemical staining with anti–Iba-1 antibody in SN 3 days after MPTP treatment shows increased microglial activation in the GR^Cx30CreERT2^ mutants. Scale bar, 50 μm. **b**, **c** Quantification of surface area (s.a) and density (right) of Iba-1 + microglia in SN (**b**) and striatum (ST) (**c**) of controls mice and GR^Cx30CreERT2^ mutants 3 and 7 days after saline or MPTP injection. **p < 0.01; *p < 0,05 controls vs mutants, post-hoc Bonferroni/Dunn test. #*p* < 0.05; ##*p* < 0.01; ##*p* < 0.01saline vs MPTP. **d**, **e** Quantification of surface area (s.a) and density (right) of GFAP + astrocytes in SN (**d**) and striatum (**e**) in control mice and GR^Cx30CreERT2^ mutants 3 and 7 days after saline or MPTP injection. #*p* < 0.05 saline vs MPTP. In all, error bars represent SEM. *n* = 5 mice/group

Acute MPTP intoxication induces glial reactivity in striatum and SN, thus we analyzed the cell density and the surface area (indicative of hypertrophy) of Iba-1 + and GFAP + cells in these regions. MPTP intoxication in SN of control mice caused a transient increase (40%) in microglial surface area at 3 days compared to saline-injected mice, thereafter decreasing to 18% at 7 days. In the astrocytic GR mutant SN, this increase was stronger (66%) at 3 days and remained elevated at 7 days (63%) (Figs. 3a, b). In the striatum of control mice, there was no change in microglial surface area after MPTP treatment whereas in the mutants a 40% increase was observed at 3 days and 105% increase at 7 days. The microglial density increased at 3 days in SN and striatum following MPTP intoxication regardless of the genotype. Notably, at 7 days, the increase remained significant in the striatum of MPTP-treated mutants compared to saline treatment (Fig. 3c). On the other hand, GFAP + astrocyte cell density and hypertrophy were similar in control and mutant mice following MPTP treatment (Figs. 3d, e).

### Profile of changes in gene expression in SN of astrocytic GR mutants following MPTP-induced injury

**Fig. 4:**
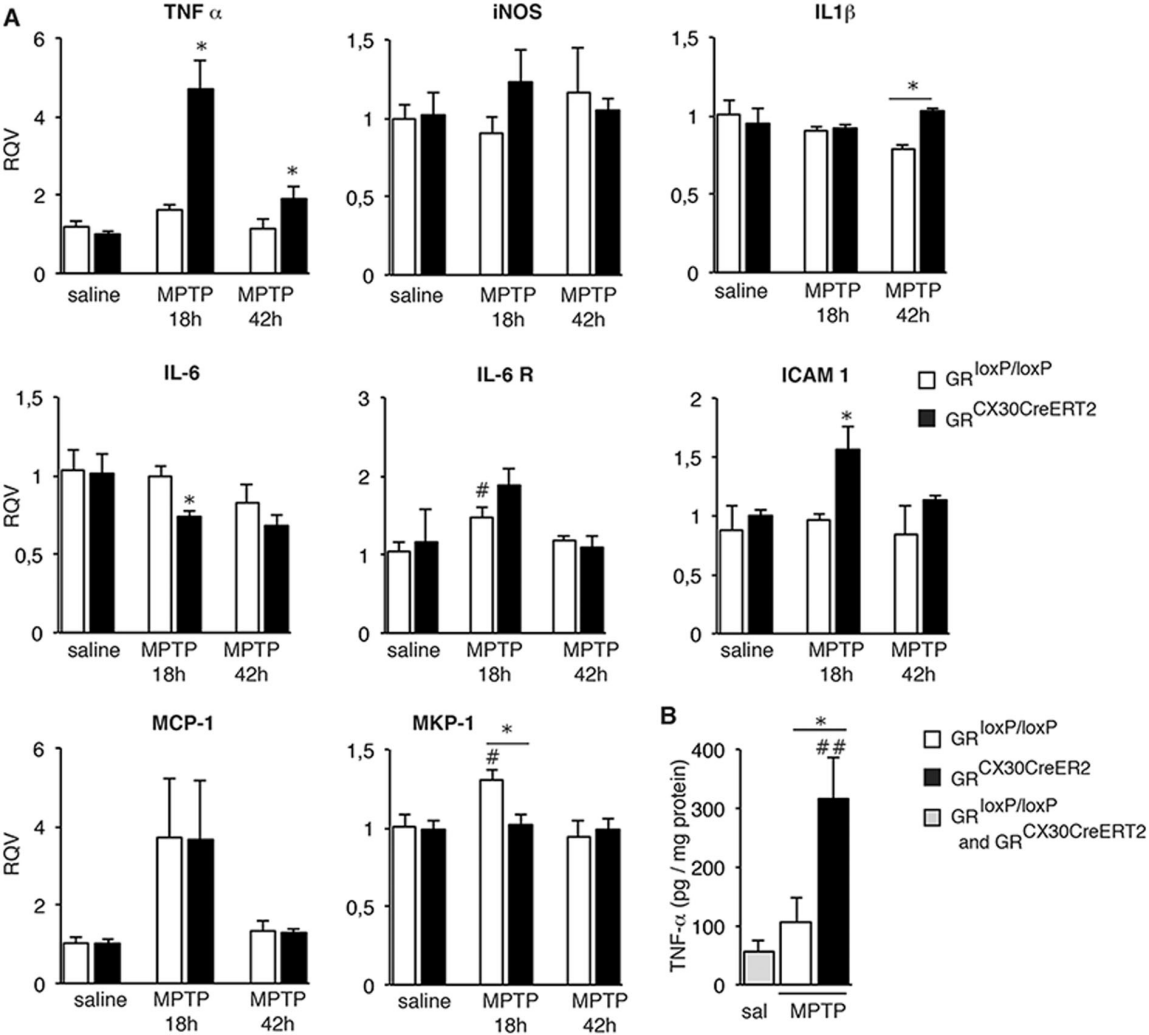
Analysis of inflammatory mediators in SN following MPTP intoxication displaying differential changes in astrocytic GR mutants compared to controls **a** Expression levels of selected inflammatory genes measured by RT-qPCR in SN of GR^Cx30CreERT2^ mutant mice compared to control mice 18 and 42 h after saline or MPTP injection. *HPRT* was used as internal control *p < 0.05 controls vs mutants; #*p* < 0.05 saline vs MPTP injected; error bars are SEM. *n* = 5 mice/group. **b** Protein level of TNF-α in SN of control and GR^Cx30CreERT2^ mutant mice 3 days after saline or acute MPTP injections. ##*p* < 0.02 saline vs MPTP injected, *p < 0.05 controls vs mutants MPTP. Error bars represent SEM. *n* = 4 mice/group

GR regulates transcription of both pro- and anti-inflammatory genes [21–24]. The elevated microglial reactivity in GR^Cx30CreERT2^ mutants prompted us to analyze the expression of GR-regulated inflammatory genes. In the SN of control and mutant mice injected either with saline or MPTP, RT-qPCR analysis at 18 and 42 h showed stronger increases in 3 pro-inflammatory genes in mutants compared to controls after MPTP: a) *TNF-α* level was higher by 4.7 fold at 18 h and remained significantly elevated at 42 h (Fig. 4a). Accordingly the TNFα protein level in the SN, 3 days following MPTP intoxication, was 2.5 fold higher in mutants (Fig. 4b), b) *ICAM-1* level was 1.4 fold higher and c) *IL-1β* remained high at 42 h.

*MCP-1* (*CCL-2*) was significantly high at 18 h regardless of the genotype (Fig. 4a). MPTP likely stimulates IL-6 signaling as *IL-6R* expression was increased in controls (Fig. 4a) whereas *IL-6* was down regulated in mutants compared to controls. *MKP-1* (mitogen-activated protein kinase phosphatase 1) whose expression is stimulated by GR was upregulated in controls and not mutants (Fig. 4a), suggesting GR regulation of p38 kinase activity through MKP-1 expression [25].

Gene expression of other functional categories was also analyzed (Fig. S1). There was no change in *glutamine synthetase* or *GLT-1* expression in either controls or mutants suggesting that MPTP treatment and GR in midbrain astrocytes do not influence glutamate regulation. Interestingly, in MPTP intoxicated mutants, the expression of *APOE* was reduced whereas *Cyp27A1* (sterol 27-hydroxylase) increased indicating cholesterol metabolism is modulated by decreased GR activity in astrocytes. We also analyzed mRNA levels of the enzymes involved in glutathione (γ-glutamylcysteinylglycine or GSH) biosynthesis: principally catalytic subunit of glutamate-cysteine ligase (γ-glutamylcysteine synthetase) (GCLC), its modulatory subunit (GCLM) and glutathione reductase. The results showed a decrease only in the *GCLM* level 42 h after MPTP administration (Fig. S1).

### Role of GR in regulating Cx30 and Cx43 expression and astrocyte hemichannel activity

TNF-α and IL-1β induce opening of astrocytic hemichannels composed of connexin-43 (Cx43) [26, 27]. To examine the role of GR in Cx functions, we first analyzed mRNA and protein levels of two major astrocytic Cxs: Cx30 and Cx43 [28] in the SN of control and GR^Cx30creERT2^ mutant mice after MPTP treatment. RT-qPCR analysis showed a strong increase of *Cx43* mRNA levels in mutants at 42 h compared to controls (Fig. S2A) with no change in *Cx30* mRNA levels (Fig. S2B). Protein levels of Cx43 and Cx30 in SN were higher in saline-injected mutants indicating that ablation of astrocytic GR enhances the basal levels of these proteins. MPTP intoxication augmented Cx30 levels in control SN, however in mutants the Cx levels were reduced (Fig. S2C). Pro-inflammatory cytokines and inflammation-activated JNK pathway were reported to decrease Cx levels in astrocyte cultures [26, 29, 30]. Phosphorylated JNK levels were increased by two-fold in the mutants (Fig. S2D), suggesting that this pathway may be involved in degrading Cx proteins in the mutants.

**Fig. 5:**
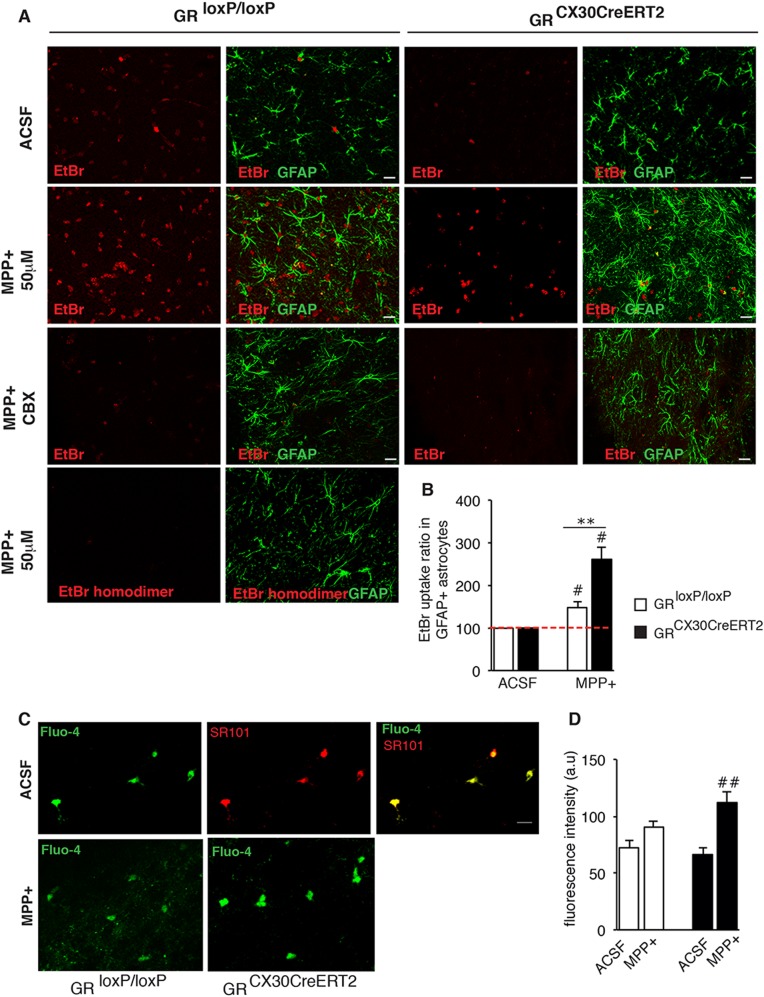
EtBr uptake and Fluo-4 fluorescence in astrocytes of midbrain slices treated acutely with MPP+ show both increased hemichannel activity and intracellular calcium alterations in astrocytes devoid of GR. **a** Representative fluorescence images showing GFAP+ astrocytes (green) and EtBr uptake (red) in acute SN slices prepared from control and GR^Cx30CreERT2^ mutant mice under control conditions (ACSF), after 2 h of treatment with MPP+ (50 μM) alone and in presence of either the general connexin channel blocker carbenoxolone (200 μM) or EtBr homodimer. Scale bar, 5 μm. **b** Quantification of EtBr fluorescence intensity in GFAP+ astrocytes normalized to control ACSF condition (taken as 100) in GFAP+ astrocytes of control and GR^Cx30CreERT2^ mutant mice after 2 h of MPP+ treatment. #*p* < 0.05 MPP+ vs ACSF, **p < 0.01 control vs mutant mice, error bars represent SEM. *n* = 3–4 mice/group. **c** Representative fluorescence images of Fluo-4 fluorescence in astrocytes, which was verified by sulphorhodamine (SR101) (upper panel) and after MPP+ treatment in control and mutant SN slices. **d** The Fluo-4 fluorescence, indicative of [Ca^2+^]_i_ was quantified in control and mutant MPP+ midbrain slices. ##*p* < 0.02 mutant ACSF vs mutant MPP+, error bars represent SEM, *n* = 3 mice/group

To examine the impact of GR and MPTP on Cx function, we assessed hemichannel activity by measuring ethidium bromide (EtBr) dye uptake [31] in the nuclei of SN astrocytes of control/mutant midbrain slices, either untreated (ACSF oxygenated medium) or treated with 50 μM MPP+ (an active metabolite of MPTP). EtBr fluorescent signal in the nuclei of GFAP+ astrocytes (Fig. 5a) in SN (control *n* = 14 slices from 3 animals, mutant *n* = 19 slices from 4 animals) was quantified. The EtBr uptake was completely inhibited by the general connexin channel blocker, carbenoxolone. Absence of EtBr fluorescence with non-permeant EtBr homodimer further confirmed that EtBr uptake reflected hemichannel activity (Fig. 5a). Hemichannel activity increased in SN astrocytes of both control and mutant slices following MPP+ treatment (Fig. 5b). Importantly, two-fold increase of EtBr uptake in mutant MPP+-treated slices was observed compared to control slices (Fig. 5b).

Cx43 hemichannels are activated by an elevation of cytosolic calcium concentration [Ca^2+^]_i_ [32, 33]. The impact of MPP+ and GR deficiency on [Ca^2+^]_i_ in SN astrocytes was analyzed using the calcium indicator Fluo-4 AM preferentially taken up by astrocytes. The specificity of uptake was confirmed by co-localization of Fluo-4 with sulforhodamine 101 [34] (Fig. 5c). Quantification of Fluo-4 fluorescence intensity in astrocytes after MPP+ treatment showed a significant increase of [Ca^2+^]_i_ in GR-inactivated SN astrocytes compared to a small non-significant increase (*p* = 0.07) in controls (Fig. 5d).

### In vivo Cx43 hemichannel activity is increased due to GR loss from astrocytes and underlies the enhnaced dopamine neurodegeneration

**Fig. 6:**
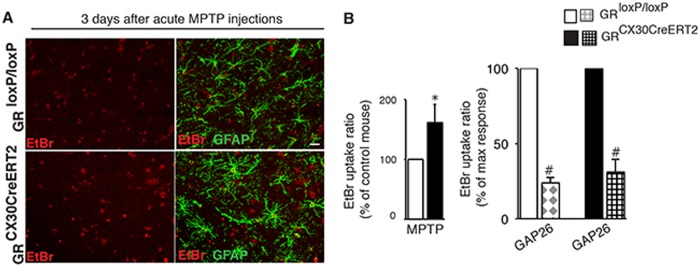
In vivo astrocytic GR regulates Cx43 hemichannel activity following MPTP intoxication. **a** Representative fluorescence images of acute midbrain slices maintained in ACSF, derived from control and GR^Cx30CreERT2^ mutant mice sacrificed 3 days after MPTP intoxication showing GFAP+ astrocytes (green) and EtBr uptake (in red). Scale bar, 5 μm. **b** (Left) Quantification of EtBr fluorescence intensity in GFAP+ astrocytes of midbrain slices prepared from GR control and mutant mice 3 days after MPTP intoxication. The change in MPTP-mutant mice is represented relative to MPTP-control mice. *p < 0.05 control vs mutant MPTP, *n* = 4 mice/group. (Right) Quantification of EtBr fluorescence intensity after 15 min of pretreatment with 200μM Gap26 mimetic peptide, an inhibitor of Cx 43 hemichannels in SN slices from controls and mutant mice sacrificed 3 days after MPTP. The results are represented relative to presence or absence of Gap26 in control or mutant slices. #*p* < 0.05, Gap26 + MPTP vs MPTP, *p < 0.05 control vs mutant MPTP, error bars represent SEM, *n* = 4/group

To show that Cx hemichannel activity observed in MPP+ -treated midbrain slices also occurs in vivo, EtBr experiments were performed on ex in vivo midbrain slices with always, in parallel, control and GR^Cx30creERT^ mutant mice injected with MPTP, sacrificed after 3 days (Fig. 6a). EtBr fluorescence in astrocytes was quantified in mutants relative to controls. In agreement with MPP+ results, a significant increase (61%) in EtBr uptake was observed in GR-inactivated SN astrocytes (Fig. 6b). Treatment of slices with Gap26 mimetic peptide, a Cx43 hemichannel inhibitor [32], significantly decreased EtBr uptake in midbrain slices from both control and mutant mice. These results indicate that GR regulates Cx43 hemichannel activity following MPTP treatment.

**Fig. 7:**
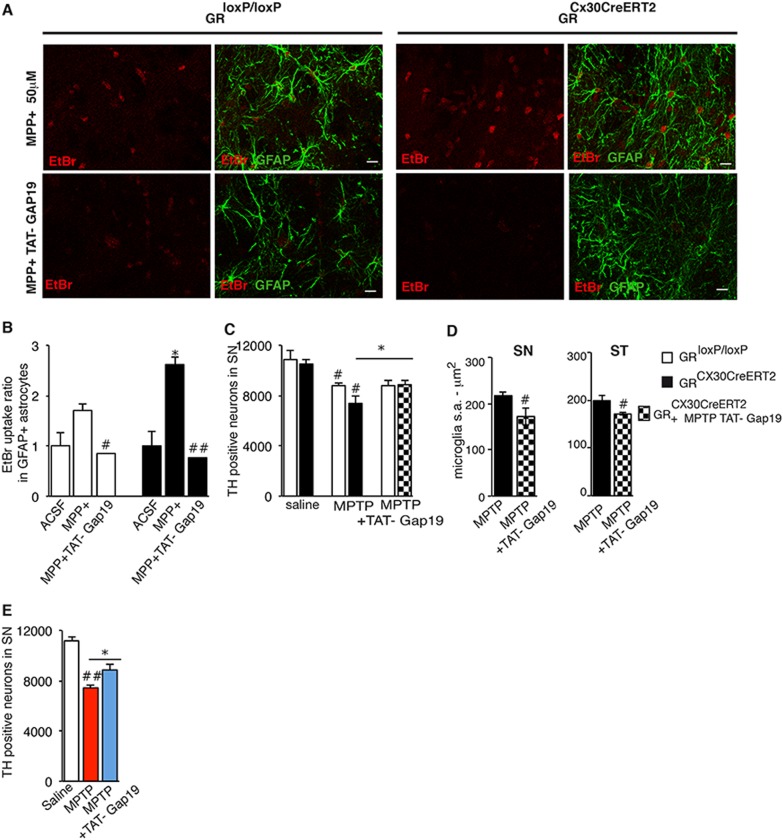
Specific inhibition of Cx43 hemichannel activity by TAT-Gap19 protects DNs and reduces microglial reactivity, particularly in astrocytic GR mutants, in response to MPTP neurotoxicity. **a** Representative fluorescence images of midbrain slices derived from control and GR^Cx30CreERT2^ mutant mice showing GFAP+ astrocytes (green) and EtBr uptake (red) after 2 h of treatment with MPP+ (50 μM), and after 30 min of pre-treatment with TAT-Gap19 (300 µM). Scale bar, 5 μm. **b** Quantitative data of experiments as illustrated in panel **a** showing almost complete inhibition of EtBr uptake both in control and mutant midbrain slices. # *p* < 0.05 control MPP+ vs controls MPP+ TAT-Gap19; ##*p* < 0.01 mutant MPP+ vs mutant MPP+ TAT-Gap19. *p < 0.05 control vs mutant slices in the presence of MPP+, error bars represent SEM, *n* = 5–6/group. **c** Quantification of TH + neurons in SN 3 days after saline or MPTP intoxication, with or without TAT-Gap19 injections. In vivo TAT-Gap19 treatment protects SN DN degeneration in GR astrocyte mutant mice. #*p* < 0.05 saline vs MPTP; *p < 0.05 mutant MPTP vs mutant MPTP TAT-Gap19, error bars represent SEM, *n* = 5 mice/group. **d** Iba1 + microglial surface area (s.a) in SN and striatum (ST) of control and astrocyte GR mutant mice after MPTP with or without TAT-Gap19 injections. TAT-Gap19 inhibits microglial hypertrophy in GR astrocyte mutant animals. #*p* < 0.05 MPTP vs MPTP TAT-Gap19 in mutant mice. **e** Quantification of TH + neurons 7 days after MPTP intoxication in the SN of C57BL/6 mice injected with saline (sal), MPTP  with ot without TAT-Gap19. *p < 0.05 MPTP + TAT-Gap19 vs MPTP in C57BL/6 mice, error bars represent SEM, *n* = 4–5 mice/group

The effects of astrocytic GR on Cx hemichannels led us to investigate the specific involvement of Cx43 hemichannels in MPTP-Parkinsonian model. TAT-Gap19 peptide, which specifically inhibits Cx43 hemichannels without inhibiting Cx43 gap junction communication or Panx1 channels, was used [35]. The peptide completely reversed MPP+ -induced EtBr uptake in SN astrocytes of control and mutant midbrain slices indicating the specific role of Cx43 hemichannels (Figs. 7a, b).

To show the role of Cx43 hemichannel activity in DN degeneration, we injected mice with TAT-Gap19 peptide (23 mg/kg), which crosses the blood brain barrier [35]. DNs in SN were quantified 3 days after saline or MPTP with or without TAT-Gap19 treatment. At this time point, TAT-Gap19 had no evident effect on survival of DNs in controls, however it completely prevented the additional loss of DNs in mutants (Fig. 7c). Moreover, microglial hypertrophy in SN and striatum decreased in TAT-Gap19 treated GR^Cx30CreERT2^ mutant mice (Fig. 7d). As hemichannel activity was observed in controls (see Fig. 5b), TAT-Gap19 peptide treatment in C57Bl/6 mice injected with MPTP was examined after a protracted time period of 7 days when 41% DN loss is observed (see Fig. 2b). TAT-Gap19 peptide reduced the loss of DNs in MPTP treated GR-intact animals (Fig. 7e). Overall these results indicate that block of Cx43 impacts microglial activation and DN loss in MPTP-Parkinsonian mice.

### GFAP and GR labeling reveals reduction in astrocytes with nuclear GR expression in SN of PD patients compared to control subjects

**Fig. 8:**
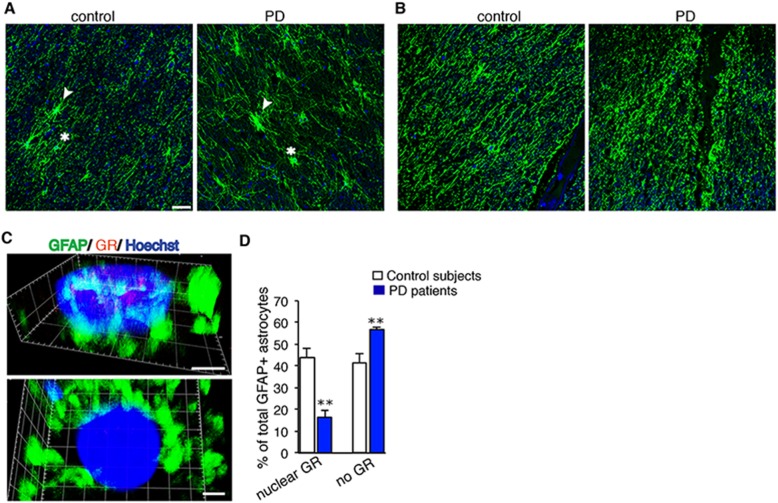
GFAP astrocytes analyzed for GR expression in SN post-mortem from control subjects and PD patients. **a**, **b** Representative fluorescent images of GFAP-labeled astrocytes in SN of paraffin sections from control subjects and PD patients. Arrowheads and asterisks in (**a**) depict two similar astrocytes in control and PD, however they are hypertrophied in PD. In (**b**) note swollen varicosities of astrocyte processes in PD compared to control. Scale bar, 10 μm. **c** 3D confocal images of GR localization in nucleus or its absence in nucleus in GFAP- labeled astrocytes. Scale bar, 2 μm. **d** Quantification of GR localization in nucleus or its absence in astrocytes, represented as % of total number astrocytes analyzed. **p < 0.01 control subjects (*n* = 6) versus PD patients (*n* = 5), error bars represent SEM

To examine whether astrocytic GRs are affected in PD, we undertook double-immunofluorescence analysis of GFAP and GR in the SN of aged-matched controls and PD patients showing Lewy body pathology in the brain stem. GFAP staining of paraffin sections revealed fine morphological features of astrocytes (Fig. 8a) and strikingly swollen varicosities of astrocytes processes were observed in some fields of SN in PD sections (Fig. 8b). Quantification of hypertrophied GFAP+ astrocytes, found both in the control and PD samples, (Fig. S3) revealed 29 ± 8/mm^2^ and 60 ± 3/mm^2^ respectively (*p* < 0.02); however in PD not all fields had high numbers of hypertrophied astrocytes (Fig. S3, bottom panel*)*.

GR was co-localized within Hoechst labeled nucleus of GFAP+ astrocytes, however there were GFAP+ astrocytes without GR in both control and PD samples (Fig. 8c). Quantification revealed 43.9 ± 4.6% of GFAP+ astrocytes with nuclear GR staining in control samples compared to 16.4 ± 3.4% for PD samples indicating that number of astrocytes with nuclear GR expression is significantly reduced in PD patients. GR expression was absent in 41.7 ± 3.4% of astrocytes in control samples compared to 56.8 ± 1.2% in PD samples implying that during PD pathology significantly more astrocytes have no GR (Fig. 8d). Taken together, these findings suggest that GR transcription regulation is likely compromised in PD patients.

## Discussion

In this study, we dissected how GR signaling in astrocytes protects DNs in SN and dopaminergic nerve terminals in the striatum during degeneration of DNs triggered by MPTP. We also examined whether GR expression is modified in post-mortem SN of PD patients. Our present results support the notion that impaired GR activity can deregulate astrocyte functions during neurodegeneration, contributing to chronic inflammation and DN loss.

### Absence of astrocytic GR increases DN loss, microglial reactivity and expression of inflammatory mediators

Increased DN loss in GR^Cx30creERT2^ mutant mice after MPTP-mediated injury paralleled stronger microglial reactivity and expression of pro-inflammatory mediators. Thus elevated TNF-α as well as *pro-IL-1β* and *I-CAM1* mRNA levels were observed in mutant SN after MPTP treatment. The high levels of TNF-α and IL-1β in mutants could potentially trigger further DN loss. The augmented hypertrophy of microglia also suggests that GR in astrocytes dampens microglial reactivity during dopamine neurodegeneration. Reactive astrocytes have been shown to exert both stimulatory and inhibitory effects on microglial activation. Thus, for example, in vitro TGF-β secreted from astrocytes was shown to curb microglial production of TNF-α and reactive oxygen species [36] whereas evidence from in vivo PD animal models and in vitro astrocyte-microglia cultures indicates that IL-1β, TNF-α, I-CAM1 or ATP released from astrocytes can enhance microglial activation [37, 38]. Concerning the latter, evidence from in vivo PD animal models and in vitro astrocyte-microglia cultures indicates that these factors released from astrocytes can enhance microglial reactivity through their respective microglial receptors [37, 38]. ICAM-1 positive astrocytes surrounded by microglia expressing LFA-1 (lymphocyte function-associated antigen) receptors have been detected in areas of intense DN loss and extracellular accumulated neuromelanin in the post-mortem SN of PD patients [11]. Other well-known GR targets genes such as iNOS or CCL-2 were not modulated in mutant mice, in contrast to our previous findings of their increased levels after inactivation of GR microglia [17, 39]. This indicates cell-specific impact of GR signaling on gene expression.

GR stimulates directly transcription of anti-inflammatory genes such as MKP-1 whose protein product dephosphorylates p38 kinase and thereby prevents stimulation of JNK and AP-1 transcription. The increased MKP-1 expression in MPTP-treated control but not mutant mice suggests that MKP-1 levels are blunted by the absence of GR in astrocytes. A decrease in MKP-1 is known to lead to increased phosphoJNK levels as seen in SN of astrocytic mutant mice after MPTP treatment. High levels of phosphoJNK and pro-inflammatory cytokines may also explain a decrease in Cx30 and Cx43 protein levels in SN of astrocytic GR mutants, as shown previously [26, 30]. However, Cx levels in astrocytes are up or down regulated by many factors such as neuronal activity or type and duration of insult [40, 41].

### Control of Cx43 hemichannel activity by astrocytic GR and its impact on dopamine neurodegeneration

The increased pro-inflammatory mediators observed in SN of GR^Cx30creERT^ mutant mice could potentially act to induce not only DN loss and microglial reactivity, but also play a role in opening astrocyte Cx hemichannels. In our previous study, TNF-α and IL-1β from hippocampal astrocytes activated Cx43 hemichannels without affecting their gap-junction communication [27]. In astrocytes of control MPP+-treated midbrain slices, we found Cx hemichannel activity upregulated, as assessed by EtBr uptake, which was increased further and significantly in GR mutant MPP+ slices. This result was then recapitulated in vivo in acute slices prepared from astrocytic GR mutant and control mice injected with MPTP.

To show the specific involvement of Cx43 hemichannels in astrocytes of SN after MPP+ or MPTP treatment, we took advantage of specific properties of Gap26 and TAT-Gap19 Cx inhibitory peptides. Gap26 reproduces a sequence of the first extracellular loop which results in the blocking of hemichannels within minutes [42], while Gap19 represents a short sequence present in the L2 domain of the intracellular cytoplasmic loop of Cx43 [43]. Its specificity relates to distinct outcomes of interactions between the C-terminal tail with the intracellular loop of Cx43 [44]. Gap19 binding to the C-terminal tail results in inhibition of hemichannel opening triggered by, for example, intracellular Ca^2+^ elevation in the range of 500 nM or below [33, 44]. Our results showed that both these peptides inhibited almost completely the hemichannel activity in SN astrocytes of mice intoxicated with MPTP. The opening of Cx43 hemichannels is also associated with increased intracellular Ca^2+^ [33, 45]. We observed a rise in [Ca^2+^]_I_ in SN astrocytes of mutant slices treated with MPP+ which suggests that together with TNF-α and Il-1β, significant Cx43 hemichannel opening may produce intercellular Ca^2+^ wave propagation thereby recruiting other reactive astrocytes and spreading inflammation and neurodegeneration-associated signals.

Importantly, we found in astrocytic GR mutant mice, Cx43 hemichannel activity impacts directly DN loss and microglial reactivity. Thus TAT-Gap19 co-treatment with MPTP intoxication in astrocytic GR mutant mice reversed both increased DN loss as well as the microglial reactivity. In wild-type mice, we observed a partial protective effect of around 20% with TAT-Gap19, which likely reflects the lower Cx43 hemichannel activity in the presence of GR. Thus astrocytic GR regulates Cx43 hemichannel activity during MPTP-induced Parkinsonism, which in turn affects dopamine neurodegeneration.

### Astrocytes changes and astrocyte GR signaling in SN of PD patients

Our results with GFAP labeling of astrocytes in sections from autopsied brains of control subjects and PD patients show greater hypertrophied astrocytes as well as swollen varicosities in astrocyte processes in some confocal fields of SN in PD. However, further detailed analyses in SN, for example, astrocytes around blood vessels versus dying neurons, should shed insights into their functional alterations in PD pathology. In PD patients, the number of astrocytes showing GR in the nucleus is drastically reduced whereas the number without GR is significantly increased. This overall GR decrease suggests that astrocytic GR functions in PD are compromised which would lead to pro-inflammatory state and neurodegeneration.

Impaired GR activity in immune-competent cells has been observed in diseases ranging from chronic obstructive lung disease, asthma, diabetes and cancer [46–48]. High cortisol levels and impaired immune functions are also features of aging and they likely play into neurodegeneration in PD as aging is regarded as an important risk factor in PD [49]. The reduced GR activity in astrocytes in our mouse model leads to increased levels of potent pro-inflammatory cytokines as well as microglial activation where connexin hemichannels play a crucial role. Together with findings in human post mortem SN, it is likely that in PD pathology progressive deregulation of astrocyte functions by GR has an effect on spreading and amplifying neuroinflammation contributing to neuronal degeneration.

## Materials and Methods

### Reagents and antibodies

MPTP, tamoxifen, MPP+, normal goat serum (NGS), diaminobenzidine, H_2_O_2_, carbenoxolone and ethidium bromide were purchased from Sigma-Aldrich. Vectastain Avidin-biotin peroxidase ABC kit, Vectashield and Vectamount mounting media were purchased from Vector. RNeasy Lipid Tissue Mini Kit was purchased from Qiagen. Superscript III reverse transcriptase kit and mouse ELISA TNF-α kit were purchased from InVitrogen. Iba-1 antibody was obtained from Wako. S100-beta and GFAP antibodies were purchased from Sigma-Aldrich; tyrosine hydroxylase (TH) and NeuN antibodies from Merck Millipore; GR antibodies from Santa Cruz (M20) and AbCam. Secondary biotinylated antibodies were purchased from Vector, whereas fluorescent secondary antibodies, anti-mouse Alexa 488, anti-rabbit Cy3 and anti-rabbit Alexa 633 were purchased from InVitrogen. Cx43-mimetic peptide Gap26 and  TAT-Gap19 were obtained from Pepnome Inc. and synthesized at >95% purity.

### Mice

All the animals were bred and raised under a 12/12 h light/dark cycle, temperature was 22 ± 1 °C and humidity 60 ± 5%. Food and water were supplied ad libitum. Experiments were performed in accordance with French (Ministère de l’Agriculture et de la Forêt, 87848) and European Community council directives of 2013 (2010/63/EU) guidelines for the care of laboratory animals and approved by University Pierre et Marie Curie and ICM committees for animal care and use. All efforts were undertaken to minimize suffering and minimal numbers of animals were used.

#### Generation of mice with conditional inactivation of GR (Nr3c1) gene in astrocytes

GR^Cx30CreERT2^ mice were produced by crossing C57BL/6 mice harboring a conditional GR (*Nr3c1)* allele [19] with mice expressing CreERT2 under control of the Cx30 promoter Tg [(Gjb6-cre/ERT2)53-33Fwp; MGI: 4420273] already bred on a C57BL/6 background [18]. Experiments were performed on male GR^loxP/loxP^;Tg(Gjb6-cre/ERT2) mice, thereafter denominated GR^Cx30creERT2^ and their control littermates (GR^loxP/loxP^) mice, which were generated by crossing GR^Cx30creERT2^ males with GR^loxP/loxP^ females. The mice were genotyped for the presence of Cre transgene [18] and GR^loxP^ alleles by PCR of tail biopsies. Both control and mutant mice were injected i.p. with 1 mg tamoxifen (solubilized in 1:9 ethanol/sunflower oil followed by 10 min bath sonication) twice daily for 5 consecutive days. The experiments were undertaken 3 weeks after the last tamoxifen injection to ensure the absence of GR protein and of tamoxifen.

#### Generation of mT/mG ^Cx30C reERT2^ for Cre recombinase verification in astrocytes

To verify Cre-mediated recombination, Tg(Gjb6-cre/ERT2) mice were crossed with a global double-fluorescent Cre reporter mice (mT/mG) [50] expressing membrane-targeted tandem dimer Tomato (mT) prior to Cre-mediated excision and membrane-targeted green fluorescent protein (mG) after Cre excision (mT/mG^Cx30 creERT2^ mice) under the control of Cx30 promoter. For Cre verification, mutant mice were injected either with tamoxifen or vehicle solution.

### MPTP treatment

All experiments were performed  on 3–5 months old male mice. GR^Cx30creERT2^ mutant mice and their control littermates GR^loxP/loxP^ were given four i.p. injections of 18 mg/kg MPTP·HCl or the same volume of saline at 2 h intervals. Mice were sacrificed at indicated time points after the last injection.

### TAT-Gap19 in vivo administration

To examine whether Cx43 is implicated in DN loss, TAT-Gap19 Cx43 inhibitor peptide was injected i.p. in parallel with MPTP at 23 mg/kg concentration in control and GR^Cx30CreERT2^ mutant mice, as well as C57BL/6 mice; total of 4 injections at a 2 h interval, and once/day for further 2 days following MPTP intoxication. The mice were sacrificed 3 or 7 days thereafter.

### Tissue preparation and staining specificity

Mice were anesthetized with CO_2_ and perfused transcardially with ice-cold 0.1 M sodium phosphate buffer (PBS) followed by ice-cold 4% paraformaldehyde (PFA) in 0.1 M sodium phosphate buffer. Brains were rapidly removed from the skull, post-fixed for 24 h in fresh 4% PFA/PBS solution and serially cut as 30 µm thick coronal sections as described [17]. The sections were stored in PBS containing 0.4% sodium azide at 4 °C until use.

#### Immunostaining

Immunohistochemistry protocols used were essentially as described in Ros Bernal et al. [17] and Carrillo-de Sauvage et al. [39]. Briefly, SN, striatal and cortical sections at 180 µm interval were incubated with either rabbit polyclonal anti-Cre (1:1000), or rabbit polyclonal anti-Iba1 (1:750) or anti-GFAP (1:2000) or mouse monoclonal anti-TH (1:1000) primary antibodies for 24 or 48 h at 4 °C with constant shaking. Sections were incubated for 2 h at room temperature with appropriate anti-mouse or anti-rabbit biotinylated secondary antibodies (1:500). Antibody detection was performed with avidin-biotin peroxidase ABC Vectastain kit according to manufacturer’s instructions and using the chromogen diaminobenzidine (DAB) as peroxidase substrate. Sections were mounted on super-frost plus slides and dehydrated in graded ethanol series and xylene, then cover-slipped using Vectamount.

For immunofluorescence, primary antibodies used were anti-Iba1 (1:750), anti-GFAP (1:2000), mouse monoclonal anti-S100β (1/200), rabbit polyclonal anti-GR (1/750) and mouse monoclonal anti-NeuN (1/500) antibodies. Appropriate secondary antibodies were anti-rabbit Cy3, anti-mouse cy3, anti- mouse or rat Alexa 488 at 1/400 dilution. After incubating PBS solution containing Hoechst (1/2000) they were mounted using Vectashield for examination and analysis by fluorescence or confocal microscope.

#### Quantification of immunostained sections

To calculate Cre recombination efficacy, sequential images from processed Z-stacks comprising four different fields per section were obtained with Zeiss microscope (Axiovert 200 M) ×40 objective, equipped with Apotome module. The number of astrocytes double stained with S100β and GR, as well as the numbers of neurons double labeled with NeuN and GR were compared between control and GR astrocyte mutant mice.

DN quantification was performed stereologically as previously described [17] on regularly spaced DAB sections of mesencephalon covering the whole SN (from rostral pole of the SN to the locus coeruleus) by bright-field microscopy using Nikon microscope ×20 objective equipped with a semiautomatic stereology system (Mercator software; Explora Nova VisioScan T4.18 system). The genotype of mice was unknown to the investigator at the time of quantification. Surface area and number of activated Iba-1 positive microglia and GFAP-labeled astrocytes were quantified from 5-6 fields chosen at random of each DAB labeled SN section (×40 objective) and covering entire SN and striatum of control and GR astrocyte mutant mice using ImageJ software. The density of striatal dopaminergic terminals was quantified based on optical density of TH-IR in striatal sections from control and mutant mice using MCID^TM^ Analysis system

### RT–quantitative PCR (RT-qPCR)

Mice were euthanized 18 or 42 h after acute MPTP treatment and their brains snap-frozen in isopentane at −25 °C. The SN region was dissected by punch at −5 to −10 °C under sterile conditions. Total RNA was prepared using the lipid RNeasy Lipid Tissue Mini Kit. To ensure complete DNA elimination, an on-column DNase step was performed. The RNA integrity and concentration were quantified based on optical density using the NanoDrop ND-1000 spectrophotometer (Thermo Fisher Scientific). cDNA synthesis was performed on 500 ng RNA, using the Superscript III cDNA Reverse Transcriptase Kit. cDNA was stored at −20 °C until use. qPCR experiments were carried out on Roche Light Cycler 480-II (Roche Diagnostic) using Syber Green master mix from Roche diagnostics. Samples for RT-PCR were run in triplicate and contained 5 μl Sybergreen, 0.3 μl of each of the forward primer and reverse primer (10 μM), and 3 μl RNase-free water. Quantification was achieved by the relative quantitation method, with serial dilutions of cDNA to construct a linear standard curve relating cycle threshold values to relative concentrations. Gene expression data were normalized to the housekeeping gene HPRT.

### TNF-α ELISA

Control and mutant mice injected with MPTP were sacrificed 48 h after the injections. Mice were decapitated, brains extracted rapidly and snap-frozen in isopentane at −25 °C. Brains were stored at −80 °C until further processing. Punches of substantia nigra were obtained from frozen tissue. SN samples were immersed in ice-cold lysis buffer containing 50 mM Tris-HCl, 100 mM NaCl, 2 mM EDTA, 1% Triton X-100 and a 1% total protease inhibitor cocktail, and sonicated for 10 s. The samples were centrifuged at 13000 rpm for 20 min at 4 °C and supernatants stored at –80 °C. Levels of the TNF-α were analyzed by an ELISA kit according to the manufacturer’s protocol. Total protein concentrations were measured using the Bradford protein assay.

### Western blot analysis

Mice were euthanized 48 h after saline or acute MPTP injections and their brains snap-frozen in isopentane. Punches of striatum and substantia nigra were lysed in 140 μl of boiling SDS 2% (with complete Roche protease inhibitor cocktail, orthovanadate 1 mM and glycerophosphate 10 mM) and then sonicated. 50 μg of protein samples were loaded on Novex NuPage 4–12% Bis-Tris gradient gels (Invitrogen). After transfer onto a PVDF membrane, the blots were probed with Cx43 and Cx30 or phosphoJNK primary antibodies. Blots were reincubated with GAPDH antibody for loading control. After incubation with secondary peroxidase-conjugated antibodies (GE Healthcare) diluted at 1:2000, signals were visualized by using the ECL detection kit (GE Healthcare).

### Hemichannel experiments

#### Acute SN slices

Acute coronal midbrain slices were prepared from 4-6 months old GR^Cx30CreERT2^ and GR^loxP/loxP^ mice. After decapitation, brains were rapidly isolated and placed in ice-cold slicing solution containing the following (in mM): 27 NaHCO_3_, 222 sucrose, 10 glucose, 2.6 KCl, 1.5 NaH_2_PO_4_, 0.5 CaCl_2_, 7 MgSO_4_ and 0.1 ascorbic acid; and bubbled with 95% O_2_/5% CO_2_. Midbrain coronal brain slices (300 μm) were cut using a vibratome (VT 1200; Leica). Slices were transferred to a holding chamber at room temperature and stabilized for 1 h in artificial CSF (ACSF) containing the following (in mM): 125 NaCl, 2.5 KCl, 25 glucose, 25 NaHCO_3_, 1.25 NaH_2_PO_4_, 2 CaCl_2_, and 1 MgCl_2_, pH 7.4 and bubbled with 95% O_2_/5% CO_2_ at room temperature in the presence of 2 mM Na pyruvate.

#### Dye uptake

Acute slices were either left untreated or incubated with different concentrations (50, 100, 200 μM) of MPP + for 1, 2 or 3 h in oxygenated (95% O_2_ and 5% CO_2_) ACSF, pH 7.4 in a chamber at room temperature. Thereafter the slices were incubated with 5 μM final concentration of ethidium bromide for 10 min. After all treatments, slices were washed ×3 with ACSF and fixed at 4 °C with 4% paraformaldehyde for 1 h. 50 μM MPP+ treatment for 2 h was found to be optimal as it induced hemichannel activity without causing non-specific membrane permeabilization as visualized by the lack of EtBr uptake. To verify for uptake via connexin hemichannels, slices were incubated 15 min prior to as well as during EtBr application with the general connexin channel blocker carbenoxolone (CBX, 200 μM), EtBr monomer (5 μM), mimetic blocking peptide Gap26 (200 μM) or TAT- Gap19 (300 μM).

#### Staining and image analysis

Slices were incubated with blocking solution (0.2% gelatin/1% Triton X-100 in PBS) for 1 h. Primary antibodies, anti-Iba1 (1/500) and anti-GFAP (1/500) were prepared in blocking solution containing 10% NGS and incubated at 4 °C overnight. The following day, slices were incubated with appropriate secondary antibodies conjugated to Alexa 488 or Alexa 633 for 2 h, then washed and mounted with Vectashield. Stacks of consecutive images at 0.5 μm intervals were acquired sequentially with three lasers at 488 and 555 and 633 nm (40×) on a confocal laser-scanning Leica microscope. Three fields were selected at random in SN per slice. 10–12 Z-projections of serial optical sections (0.5 μm) were then reconstructed with ImageJ software. Fluorescence was quantified in arbitrary units with imageJ software. Dye uptake ratio was calculated as the subtraction (F-F0) between the fluorescence F from cells and the background fluorescence F0 measured where no labeled cells were detected.

To measure hemichannel activity in SN slices prepared from control or GR^Cx30CreERT2^ mutant mice injected i.p with MPTP as above and sacrificed 3 days after, uptake of EtBr in SN slices of the mutant mouse was calculated relative to uptake in SN slices of the control mouse, processed at the same time.

### Intracellular calcium measurements

Acute brain slices from control and GR^Cx30CreERT2^ mutants incubated in ACSF with 95% O_2_/5% CO_2_, were treated with 50 μM MPP+ for 2 h at room temperature. Slices were then incubated for 45 min in dark at 37 °C in ACSF containing calcium indicator Fluo-4AM (5 μM), a calcium indicator which is preferentially loaded in astrocytes [51]. Astrocytes were further identified by their ability to uptake the fluorescent tracer sulforhodamine 101 (SR 101) [52]. After dye loading, slices were thoroughly washed by 20 min incubation in ACSF solution (equilibrated with 95% O2 to 5% CO2). They were placed on a coverslip coated with polylysine. Fluo-4 and SR 101 were excited at respectively 488 nm and 587 nm. Stacks of images were rapidly acquired through a water immersion objective on a SP5 confocal microscope with multi-photon laser. Images were analyzed with Image J software [45].

### Human postmortem sections

The samples were obtained from brains collected in a Brain Donation Program of the Brain Bank “NeuroCEB” run by a consortium of Patients Associations that include ARSEP (association for research on multiple sclerosis), CSC (cerebellar ataxias), and France Parkinson. The signed consents were either from the patients or their next of kin in their name, in accordance with the French Bioethical Laws.

The mean age of control and PD subjects was 67.5 ± 6.8 and 76.6 ± 3.8 years respectively (mean ± SEM). Control group comprised of 3 males and 3 females whereas PD group consisted of 3 males and 2 females. 2–3 transversal sections/brain covering dorsal-medial and ventral-lateral areas of SN, (SN area identified in brain stem tissue by exiting fibers of 3^rd^ cranial nerve), were used for immunofluorescence experiments. Immunofluorescence of GR and GFAP was carried out both with 10 μm paraffin sections (*n* = 2 for both control subjects and PD) as well as 30 μm fresh sections which were stored at −80 °C (*n* = 4 for controls and 3 for PD). For each experiment, equal number of control and PD sections were included for immunolabeling. To delineate SN area, TH immunohistochemistry was performed on adjacent sections. Surface area of SN comprising TH+ dopamine neurons was measured using a Nikon microscope (Eclipse, ×20 objective) equipped with a semi-automatic stereology system (Mercator software; Explora Nova VisioScan T4.18 system). The mean SN area in control and in PD samples was 29.94 ± 3.8 mm^2^ and 29.8 ± 4.0 mm^2^ per section, respectively. For GR and GFAP expression, similar results were obtained for paraffin sections and fresh tissues and thus the data were pooled. Paraffin-embedded sections were deparaffinated in a series of xylene/alcohol solutions, and antigen retrieval was achieved by heating the sections at 80 °C for 20 min in 10 mM sodium citrate buffer, pH 6.0. Cryostat fresh sections were air-dried before fixation with freshly prepared 4% PFA/ PBS solution for 20 min. The sections were rinsed in tris-buffered saline (TBS) solution containing 0.5% Triton before blocking for 2 h in 2% goat serum/TBS-0.5% Triton. They were incubated with rabbit polyclonal anti-GFAP antibody (Sigma-1/200) and mouse anti-GR antibody (AbCam-1/200) in 2% goat serum/TBS-0.5% Triton for 48 h at 4 °C. After washes in TBS-0.5% Triton, they were incubated with secondary antibodies: Alexa goat anti-rabbit 488 and donkey anti-mouse Cy3, washed, stained with Hoechst 33342 and mounted using vectashield.

For imaging, confocal laser-scanning Leica microscope with 40 × objective was used to acquire sequentially stacks of consecutive images at 2 μm intervals with 3 lasers at 488 nm (GFAP) and 555 nm (GR) and 633 nm (Hoechst). The fluorescence across one entire image was determined; a threshold for positive staining was set for each channel and same settings were used for all acquisitions. 7 fields (each 0.15 mm^2^) spanning SN were selected at random; in total they constitute about 1/30 of total SN area of the section. For 30 μm sections, images were reconstructed to obtain 6–8 μm thickness images corresponding approximately to nuclear size, this to avoid overlapping of cells. These images of astrocytes with Hoechst staining were analyzed for GR localization and quantification using ImageJ software, ARIVIS software was used for 3D reconstruction.

### Statistical analysis

Data are expressed as mean ± SEM. Statistical analyses were performed by using Mann-Whitney non-parametric test and two-way ANOVA. Post hoc comparisons between mutant and control groups were made using a Bonferroni/Dunn test. Differences of *p* ≤ 0.05 were considered statistically significant. Statistical analyses were carried out using StatView 5.0 software.

## Electronic supplementary material

Supplementary Figure 1

Supplementary Figure 2

Supplementary Figure 3

Supplementary figure legends
