# Supplementary figures and images for: Glucocorticoid receptor in astrocytes regulates midbrain dopamine neurodegeneration through connexin hemichannel activity
Source: Cell Death Differ. 2018 Jul 13;26(3):580–96. doi: 10.1038/s41418-018-0150-3 (PMC6370798; doi:10.1038/s41418-018-0150-3)

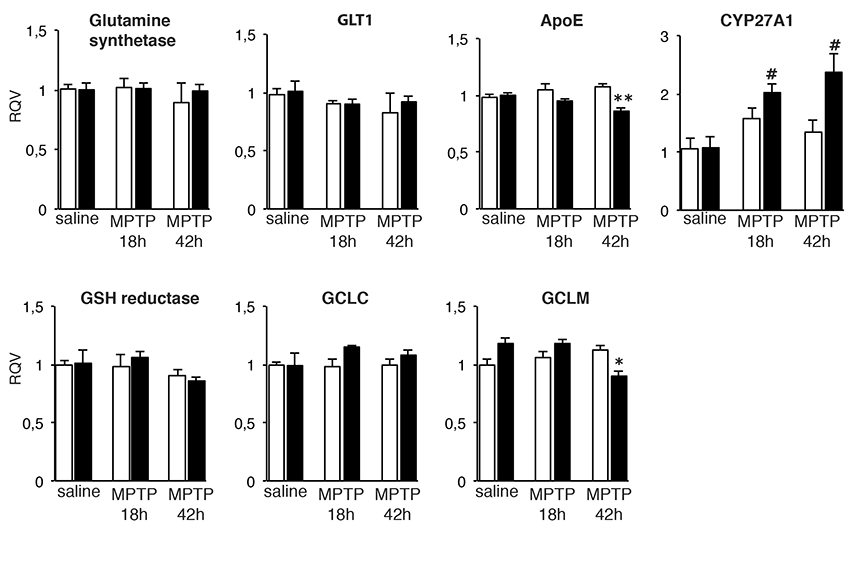

Supplement: Supplementary file 1 — Supplementary Figure 1 [file 41418_2018_150_MOESM1_ESM.tif]

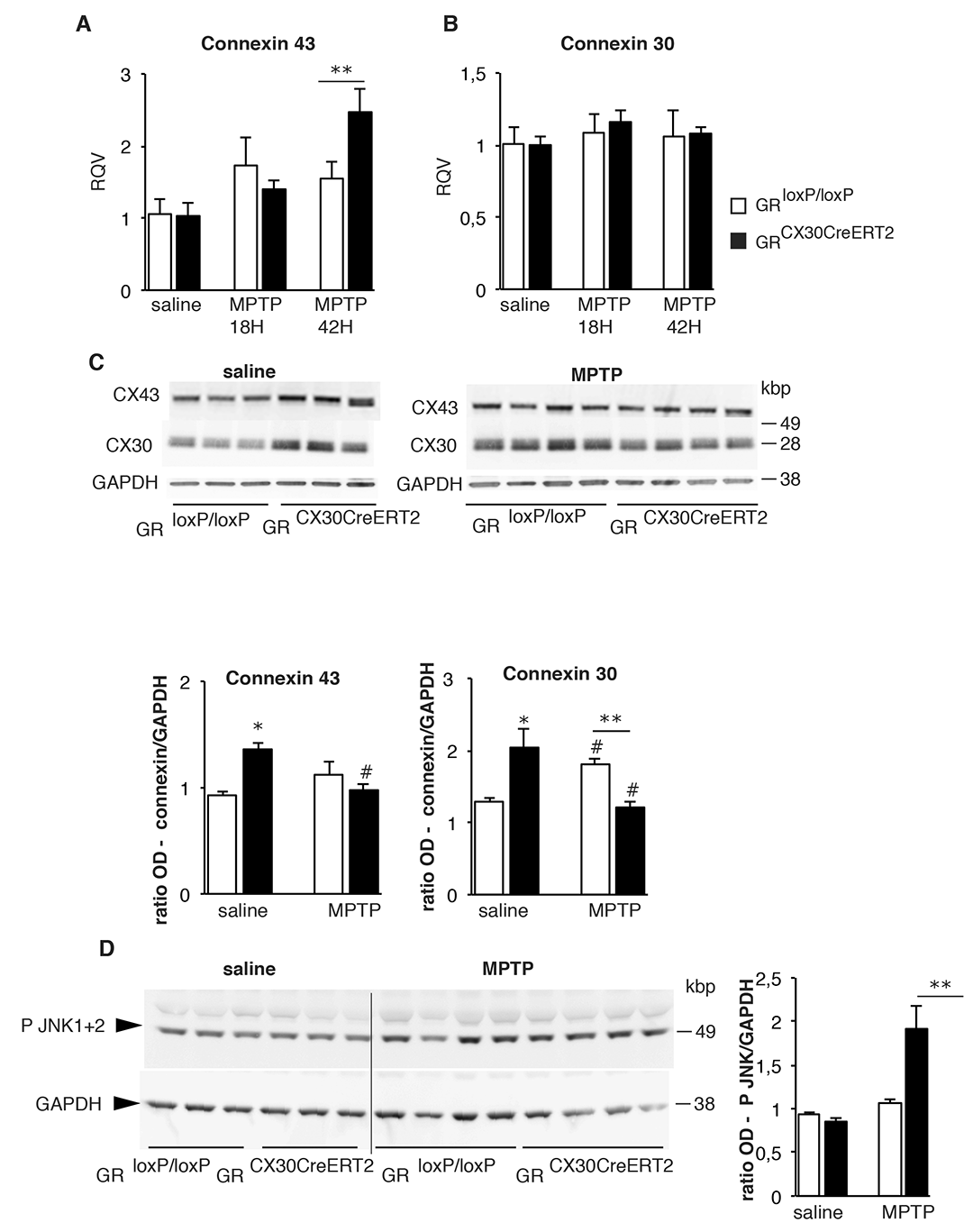

Supplement: Supplementary file 2 — Supplementary Figure 2 [file 41418_2018_150_MOESM2_ESM.tif]

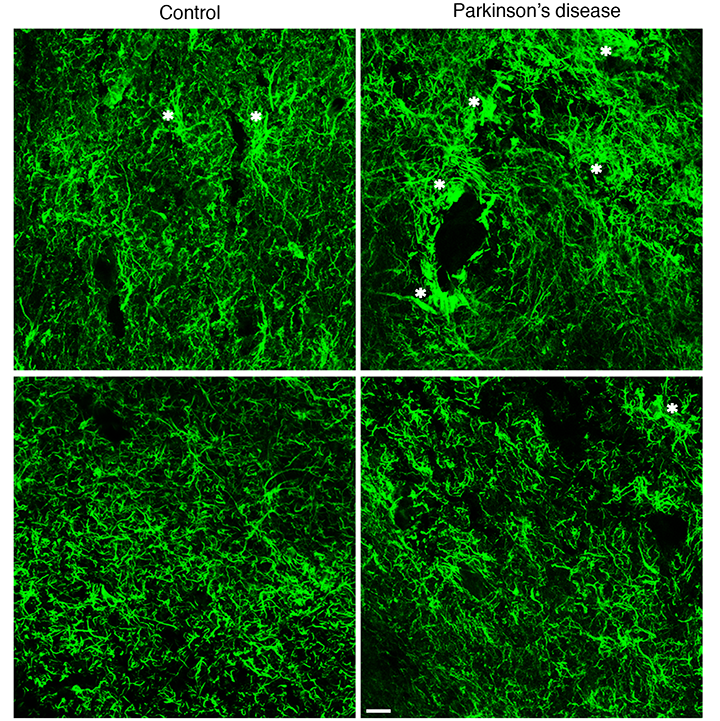

Supplement: Supplementary file 3 — Supplementary Figure 3 [file 41418_2018_150_MOESM3_ESM.tif]
