# Supplementary material for: Glucocorticoid receptor in astrocytes regulates midbrain dopamine neurodegeneration through connexin hemichannel activity
Source: Cell Death Differ. 2018 Jul 13;26(3):580–96. doi: 10.1038/s41418-018-0150-3 (PMC6370798; doi:10.1038/s41418-018-0150-3)
Supplement: Supplementary file 4 — Supplementary figure legends [file 41418_2018_150_MOESM4_ESM.docx]

**SUPPLEMENTARY INFORMATION**

**Figure S1: Analysis of astrocyte genes involved in glutamate control, cholesterol metabolism and oxidative stress in SN of astrocytic mutant GR mice after MPTP treatment**

RT-qPCR analysis of selected genes related to glutamate and cholesterol metabolism as well as oxidative stress in SN of GR^Cx30CreERT2^ mutant mice compared to control mice 18 and 42 h after saline or MPTP injections. HPRT gene was used as internal control. ** *p* < 0.02 and * *p* < 0.05 control vtant MPTP mice; # *p* < 0.05 saline vs MPTP injections; error bars represent SEM, n=5 mice/group.

**Figure S2: Cx30, Cx43 and phospho-JNK levels in SN of astrocytic GR mutants compared with control mice after MPTP treatment**

**A & B**: RT-qPCR analysis of Cx43 (**A**) and Cx30 (**B**) expression levels in SN of GR^loxp/loxp^ and GR^Cx30CreERT2^ after saline or MPTP injections. ** *p* <0.01, control vs mutant MPTP mice. n= 5/group

**C &D**: WB analysis of Cx43 and Cx30 (**C**) and phospho-JNK (**D**) levels in SN 48h after saline or MPTP injections in control and GR^Cx30CreERT2^ mice. Quantification of signals, results expressed as ratio of OD relative to GAPDH. # *p* < 0.05, saline vs MPTP. ** *p* < 0.02, control vs mutant MPTP, error bars represent SEM, n=3-4/group.

**Figure S3: GFAP labeled astrocytes in human SN from control and PD samples**

Representative images of GFAP labeled astrocytes in two SN regions of control and PD samples, taken from GFAP labeling in fresh-frozen tissue sections. Hypertrophied astrocytes (asterisks) are observed in SN of both control and PD samples. Scale bar, 5 μm.
